# Whole-exome sequencing reveals the etiology of the rare primary hepatic mucoepidermoid carcinoma

**DOI:** 10.1186/s13000-021-01086-3

**Published:** 2021-04-08

**Authors:** Ping Hou, Xiaoyan Su, Wei Cao, Liping Xu, Rongguiyi Zhang, Zhihao Huang, Jiakun Wang, Lixiang Li, Linquan Wu, Wenjun Liao

**Affiliations:** 1 Department of Hepatobiliary and Pancreatic Surgery, The Second Affiliated Hospital of Nanchang University, No.1, Minde Road, Nanchang, China; 2 Department of Pathology, The Second Affiliated Hospital of Nanchang University, No.1, Minde Road, Nanchang, China; 3 Department of General Surgery, The First Affiliated Hospital of Nanchang University, No.17, Yongwaizheng Street, Nanchang, China; 4 Department of Pathology, The Third Affiliated Hospital of Nanchang University, No.128, Xiangshan Road, Nanchang, China

**Keywords:** Hepatic mucoepidermoid carcinoma (HMEC), Somatic GNAS R201 mutation, Germline Fanconi’s anemia mutation, Whole exome-sequencing (WES)

## Abstract

**Background:**

Primary hepatic mucoepidermoid carcinoma (HMEC) is extremely rare and the molecular etiology is still unknown. The *CRTC1-MAML2* fusion gene was previously detected in a primary HMEC, which is often associated with MEC of salivary gland in the literature.

**Methods:**

A 64-year-old male was diagnosed with HMEC based on malignant squamous cells and mucus-secreting cells in immunohistochemical examination. Fluorescence in situ hybridization (FISH) was used to detect the *CRTC1-MAML2* fusion gene in HMEC. Whole-exome sequencing and Sanger sequencing were used to reveal the molecular characteristics of HMEC and analysis was performed with public data. Pedigree investigation was performed to identify susceptibility genes.

**Results:**

Hematoxylin–eosin staining and immunohistochemistry revealed that the tumor cells were composed of malignant epidermoid malignant cells and mucous cells, indicating a diagnosis of HMEC. The *CRTC1-MAML2* fusion gene was not detected in the primary HMEC, and somatic mutations in *GNAS*, *KMT2C* and *ELF3* genes were identified by sequencing. Analyses of public data revealed somatic *GNAS* alterations in 2.1% hepatobiliary tumors and relation with parasite infection. Heterozygous germline mutations of *FANCA*, *FANCI*, *FANCJ/BRIP1* and *FAN1* genes were also identified. Pedigree investigation verified that mutation of Fanconi’s anemia susceptibility genes were present in the pedigree.

**Conclusions:**

Here we provide the first evidence of the molecular etiology of a rare HMEC associated with germline Fanconi’s anemia gene mutations and somatic GNAS R201H mutation.

**Supplementary Information:**

The online version contains supplementary material available at 10.1186/s13000-021-01086-3.

## Background

Mucoepidermoid carcinoma (MEC) is a relatively common malignant neoplasm of the salivary gland that is mainly characterized by the pathological manifestation of mucous cells, intermediate cells and epidermoid malignant cells in intimately mixed nests. MEC also occurs at low frequencies at other sites, such as the esophagus, thyroid gland, lacrimal gland, lung, thymus, breast, anal canal, uterine cervix, hepatobiliary system and the pancreatic system [1]. Over 50–60% of salivary MEC (SMEC) cases are associated with a fusion gene, *CRTC1/CRTC3-MAML2*, which results from the chromosomal translocation t(11;19)(q21;p13) [2]. The *CRTC1-MAML2* fusion gene was also observed in primary cervical MEC [3] and bronchopulmonary MEC [4], which are different from adenosquamous carcinoma. However, pancreatic MEC did not show the *CRTC1/3-MAML2* fusion and was a morphologic variant of pancreatic adenosquamous carcinoma [5]. The presence of the *CRTC1-MAML2* fusion gene in primary hepatic MEC (HMEC) was controversial [6, 7]. MEC was also associated with hematopoietic stem cell transplant in Fanconi’s anemia patients [8]. So far, only 23 cases with primary hepatobiliary MEC (including hepatic, biliary and gallbladder MEC) that were pathomorphologically similar to SMEC have been reported in the literature (Table 1), but the molecular etiology of HMEC is still unknown. Here we revealed the genetic abnormality of a primary HMEC by whole-exome sequencing (WES).

**Table 1:** Basic characteristics of 23 patients with primary hepatobiliary mucoepidermoid carcinoma, including in the liver, gallbladder and biliary tract, reported in the literature

Case	Year	Area	Age (year)	Gender	Location	Metastasis	Size (cm)	Hepatitis	AFP ng/ml	CA199 u/ml	CEA ng/ml	Treatment	OS (day)	Reference
1	1971	Argentina	44	M	RL	Non	15	Np	Np	Np	Np	S	45	
2	1980	Hong Kong	65	M	RL	Y	8	Np	Np	Np	Np	Con	14	
3		Hong Kong	63	F	LL	Y	6	Np	Np	Np	Np	Con	16	
4	1982	Hong Kong	44	F	LL	Non	12	HBV	Np	Np	Np	S + C	180	49
5		Hong Kong	66	M	BD	Y	4	**Non**	Np	Np	Np	PTCD+S	7	
6		Hong Kong	62	M	BD	Non	1.5	**Non**	Np	Np	Np	S	300+	
7	1984	Japan	78	M	LL	Y	11	Np	12.5	Np	1300	C	90	42
8	1986	Krean	35	M	LL	Non	18	Np	< 5	Np	Np	Con	14	
9	1986	Australia	59	F	RL	Y	18	Np	Np	Np	Np	S	14	
10	1987	Japan	46	F	LL	Non	3	Np	20	Np	Np	S	330	30
11	1992	Italy	66	F	LL	Y	9.5	**Non**	< 5	500	< 2	S	180	41
12	1994	Krean	68	M	LL	Non	10	Np	Np	Np	Np	TACE+C	1095	
13	2000	Thailand	64	M	LL	Y	5	Np	Np	Np	Np	Con	210	
14	2003	Krean	52	M	LL	Y	7	Np	< 5	400	Np	S	180	28
15	2004	Krean	69	F	RL	Y	16	**Non**	< 5	240	Np	S	120	
16	2008	Japan	81	F	RL	Y	10	**Non**	< 5	14,893	Np	C	117	
17	2011	Krean	70	M	BD	Y	8	HCV	< 5	349	Np	R + C	106	
18	2012	Japan	68	M	BD	Y	5	**Non**	Np	50.8	Np	S + R + C	90+	29
19	2013	America	83	F	BD	Y	2	Np	Np	940	10	S + C	390	7
20	2014	China (mainland)	60	F	LL	Y	8.5	**Non**	< 5	50	Np	S + R	180	
21	2019	India	50	M	GB	Y	8	**Non**	< 5	652	77	S	180	1
22	2019	Japan	79	F	RL	Y	4	**Non**	< 5	415	146	S + R + C	3650+	6
23	2020	China (mainland)	60	M	LL	Non	13	**Non**	< 5	151	10	S	90	present

*Abbreviations*: *Non* Not have, *Np* Not provided, *RL* Right liver, *LL* Left liver, *BD* Bile duct, *GB* Gallbladder, *HCC* Hepatocellular carcinoma, *CC* Cholangiocarcinoma, *ASC* Adenosquamous carcinoma, *GBC* Gallbladder carcinoma, *S* Surgery, *R*: Radiotherapy, *C* Chemotherapy, *Con* Conservative, *TACE* Transcatheter arterial chemoembolization, *PTCD* Percutaneous transhepatic cholangial drainage, *OS* Overall survival

## Methods

### Case material

**Fig. 1:**
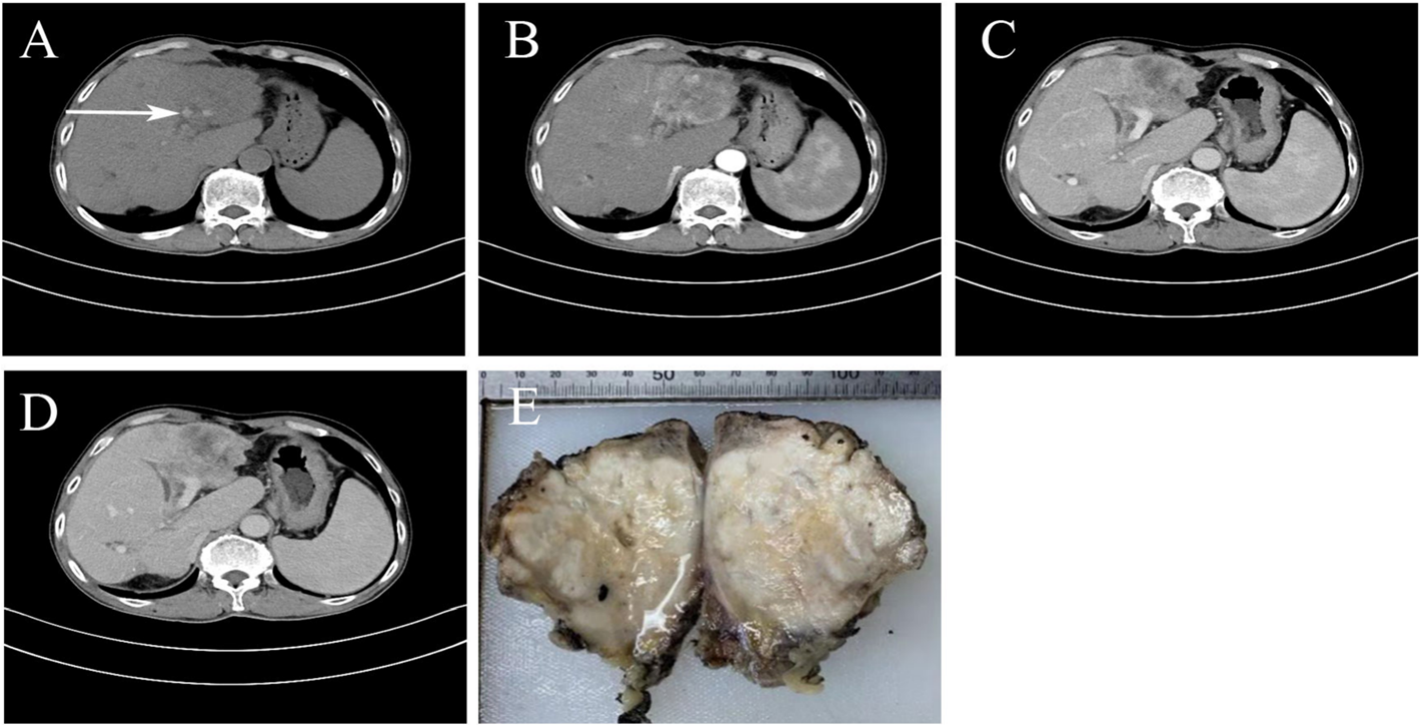
Results of preoperative CT, postoperative gross examination. **a** Abdominal non-contrast CT scan revealed a low density mass measuring approximate 10 cm in diameter at the left hepatic lobe and intrahepatic bile ducts with multiple stones (white arrow). **b** Enhanced CT scan (arterial phase) revealed a heterogeneous enhanced mass in the left lobe of the liver. **c** Enhanced CT scan (portal phase) revealed a persistent inhomogeneous enhanced mass and formation of tumor thrombus in the left branch of portal vein. **d** Enhanced CT scan (delayed phase) revealed a persistent inhomogeneous enhanced mass. **e** Gross examination of the resected liver mass indicated an irregular, white solid tumor without a fibrous capsule. The border between the tumor and normal liver tissue was indistinct

A 64-year-old male was admitted to the Second Affiliated Hospital of Nanchang University on September 16, 2019 with a complaint of repeated abdominal distension over a period of 1 year. He had a previous history of icteric hepatitis and unknown biliary tract surgery 30 years prior and coronary artery stenosis disease with an implanted stent 1 year ago. No abnormality was found in the physical examination. Most preoperative laboratory tests were in the normal range, including serum glutamic pyruvic transaminase, glutamic oxaloacetic transaminase, total bilirubin, electrolyte, glucose and platelet count and serum alpha fetoprotein level. Hepatitis B surface antigen and hepatitis C antibody were both negative. Abnormal blood test results included C-reactive protein 112.3 mg/l (normal value, < 10 g/l), white blood cell 9.82 × 10^9^/l (normal value, 3.5 × 10^9^/l), red blood cell 3.68 × 10^9^ (normal value, 4.3–5.8 × 10^9^/l), hemoglobin 103 g/l (normal value 130–175 g/l), alkaline phosphatase 483.9 U/L (normal value 45–125 U/L), γ-glutamine dehydrogenase 404.84 U/L (normal value 10–60 U/L), carcinoembryonic antigen (CEA) 10.2 ng/mL (normal value, < 5.0 ng/mL) and carbohydrate antigen 19–9 (CA19–9) 151.2 U/mL (normal value, < 37 U/mL). The abdominal non-contrast computer tomography (CT) scan revealed a low density mass measuring approximately 10 cm in diameter at the left hepatic lobe and intrahepatic bile ducts with multiple stones (white arrow) (Fig. 1a). Enhanced CT scan (arterial phase) revealed a heterogeneous enhanced mass in the left lobe of the liver (Fig. 1b); enhanced CT scan (portal phase) revealed a persistent inhomogeneous enhanced mass and formation of tumor thrombus in the left branch of the portal vein (Fig. 1c) and a persistent inhomogeneous enhanced mass in delayed phase (Fig. 1d). Ultrasonography and magnetic resonance imaging further validated the results of CT. Gastroscopy revealed chronic non-atrophic gastritis. Bone scan and chest CT were performed and excluded metastatic disease. CT scan revealed no solid mass in the head and neck, parotid gland, thyroid gland and pituitary gland (data not shown). The patient underwent resection with left hemihepatectomy and choledocholithotomy and T-tube drainage. The patient was diagnosed with HMEC based on malignant squamous cells and mucus-secreting cells in pathological examination. There was gradually increased jaundice in the postoperative term, and the patient died of hepatic function failure at postoperative 3 months.

This study was approved by the Ethics Committee of the Second Affiliated Hospital of Nanchang University, and informed consent was obtained from the patient and his family. Parts of tumor tissues and corresponding non-tumor tissues were immediately conserved in a liquid nitrogen tank at − 80 °C. Some tissue samples were fixed in 10% formaldehyde and paraffin-embedded for pathological diagnosis and immunohistochemistry. Exome sequencing and Sanger sequencing were performed in fresh tumor tissue and corresponding non-tumor tissue. The WES data from this study were deposited in NCBI Sequence Read Archive under accession SRA:SRP 266690.

### Histopathological examination

Hematoxylin-eosin staining and immunohistochemistry were performed on tumor samples and corresponding non-tumor samples following standard procedures. Primary antibodies included antibodies against MUC1 (1:200), MUC5AC (1:200), CK7 (1:250), CK19 (1:150), p63 (1:150) and CEA (1:250) (all from Leica, Wetzlar,Germany). All tissue sections were processed using the Lab Vision system (Thermo Scientific, Kalamazoo, MI, USA). The two senior pathology specialists from the Affiliated Hospital of NanChang University (XY Su and LP Xu) who examined the results suggested a diagnosis of HMEC.

### Fluorescence in situ hybridization (FISH) analysis

FISH was performed on formalin-fixed paraffin-embedded sections of HMEC using a commercially available Dual Color fusion Probe (F.01358, Anbiping Medical Laboratory, Guangzhou, China) following the manufacturer’s instructions. Green fluorescence–labeled *CRTC1/MECT1* (19p13) probe (G) and red fluorescence–labeled *MAML2* (11q21) probe (R) were used to evaluate the fusion gene. In samples harboring the fusion gene, the green signal and the red signal from probe binding generates a yellow signal (F). The normal signal mode is 2G2R and the typical positive signal mode is 1G1R2F. Histopathology was evaluated by pathologists to determine the tumor and hybridization area. FISH was performed for the *CRTC1/MECT1-MAML2* fusion gene and 200 interphase tumor cells were examined in at least two visual fields.

### WES

Exome capture was performed using 0.6 μg genomic DNA per sample. Sequencing libraries were generated using the Agilent SureSelect Human All Exon V6 kit (Agilent Technologies, CA, USA). The DNA libraries were sequenced on an Illumina HiSeq platform and 150 bp paired-end reads were generated. The original fluorescence image files were transformed to raw data by base calling and clean data were recorded in FASTQ format. Valid sequencing data were mapped to the reference human genome (UCSC hg19) by Burrows-Wheeler Aligner software [9] and results were stored in BAM format. Samtools mpileup and bcftools were used for variant calling and to identify single-nucleotide variants (SNVs) and InDel [10]. Functional annotation ANNOVAR was performed for annotation for Variant Call Format and detecting variants by 1000 Genome, dbSNP and other databases [11–13]. Given the significance of exonic variants, gene transcript annotation databases such as Consensus CDS, RefSeq, Ensembl and UCSC were also included to determine amino acid alterations [14]. Using the paired samples of tumor tissue and corresponding non-tumor tissues, somatic SNVs were detected by MuTect [15], somatic InDels by Strelka [16] and somatic CNVs by Control-FREEC [17]. SIFT, Polyphen/Polyphen2 and MutationAssessor were used to predict the impact of SNVs on function [18–20]; and truncated mutations (nonsense and frameshift) also resulted in high functional impact alterations.

### Public data analysis

The search and analytic strategy was performed using public data (http://www.cbioportal.org/) [21, 22]. SNVs were examined in hepatocellular carcinoma (HCC) and cholangiocarcinoma (CHL). We also downloaded SMEC SNV data [23] from published studies to examine the relation between the alterations in the current case with downloaded data. We used an online tool to construct Venn diagrams (https://bioinfogp.cnb.csic.es/tools/venny/).

### Sanger sequencing and pedigree survey

Purified DNA was isolated from fresh tumor tissue and corresponding non-tumor tissue from the proband and peripheral blood lymphocytes of four family members using the TIANamp Genomic DNA Kit (Beijing Biotech, China). PCR was performed using the PTC-200PC instrument (BIO-RAD, USA). Primer sequences are shown in Supplementary Table 1. The PCR products were examined using an ABI 3730XL DNA Analyzer (Applied Biosystems, USA).

## Results

### Microscopic features and immunohistochemistry

**Fig. 2:**
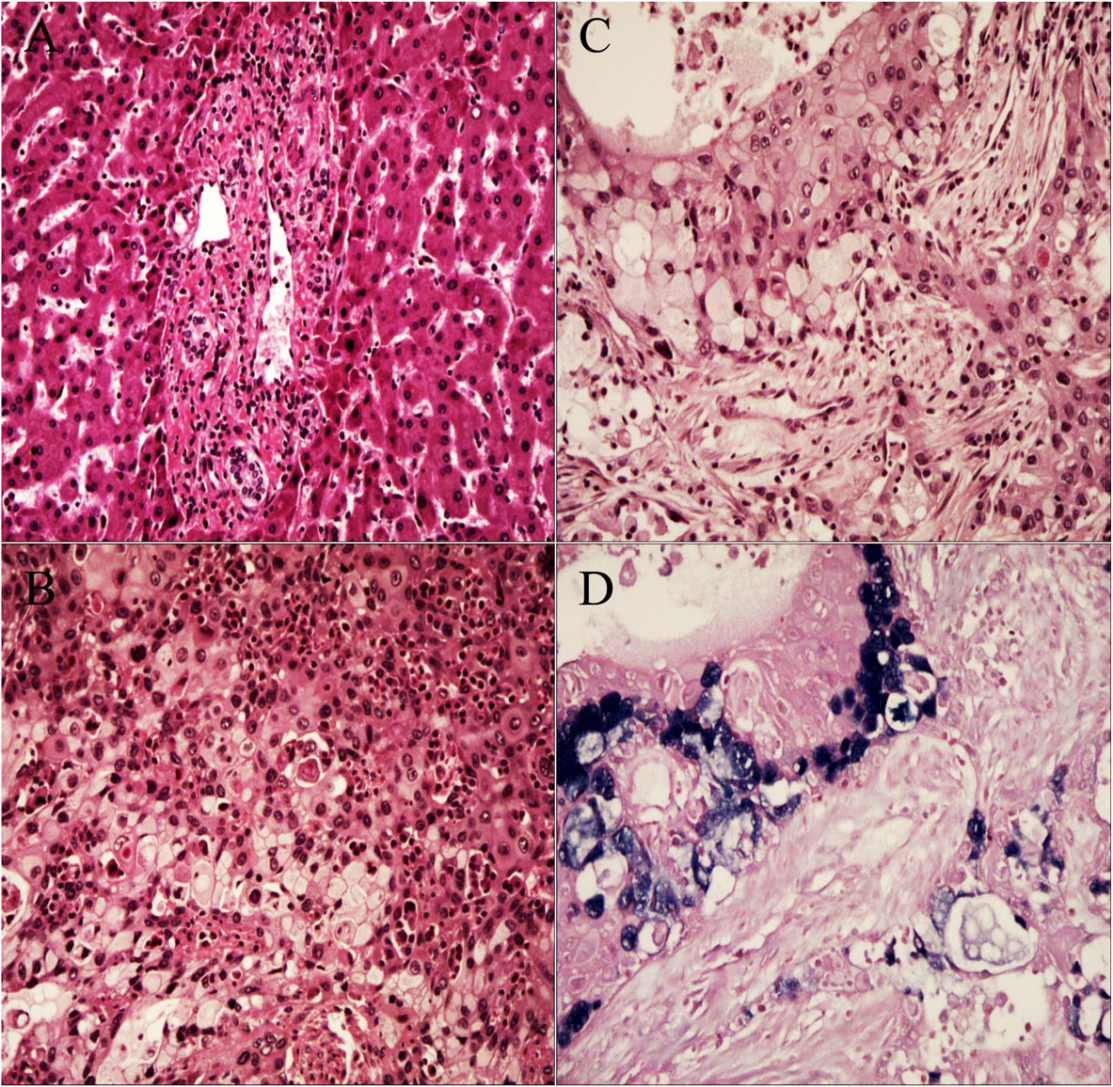
Pathological results. **a** Dystrophic arteries and a large number of inflammatory cells, mainly including lymphocyte infiltrations, were observed in the portal area (hematoxylin and eosin (H&E) staining, magnification 100×). **b** Eosinophil infiltrations were seen in the tumor area (H&E staining, magnification 200×). **c** H&E staining revealed malignant epidermoid cells and mucus-producing cells with intracytoplasmic mucin. The tumor cells infiltrated into the stroma with occasional keratinization (magnification 200×). **d** Alcian blue stain-positive material highlights the mucin in mucin-producing cells (magnification 200×)

The mass was an irregular specimen measuring approximately 10 × 7 × 6 cm, revealing no fibrous capsule and tough solid texture; resection from the middle edge showed grayish-white nodules without necrosis. Black pigment stones were also found in the small intrahepatic bile duct (Fig. 1e). The resection edges were free of tumor. There was an absence of cirrhosis in the corresponding non-tumor sample, but massive inflammatory cell infiltration was observed in tumorous and portal areas (Fig. 2a). Hematoxylin and eosin staining revealed that the tumor cells were composed of epidermoid malignant cells, mucous cells and intermediate cells (Fig. 2b). The distribution of epidermoid cells was not nest-like but was surrounded with intercellular bridges, with only a few glandular structures and occasional keratinization. Mucin-producing cells showed mainly abundant clear cytoplasm with eccentric pyknotic nuclei and predominantly showed intracellular mucus and rare extracytoplasmic mucin (Fig. 2c). The heterogeneity of epidermoid cells was obvious, with infiltrated stroma and invasion, but nerve, lymphovascular, blood invasion and necrosis were not apparent*.* Alcian blue staining revealed mucin in mucin-producing cells (Fig. 2d).

**Fig. 3:**
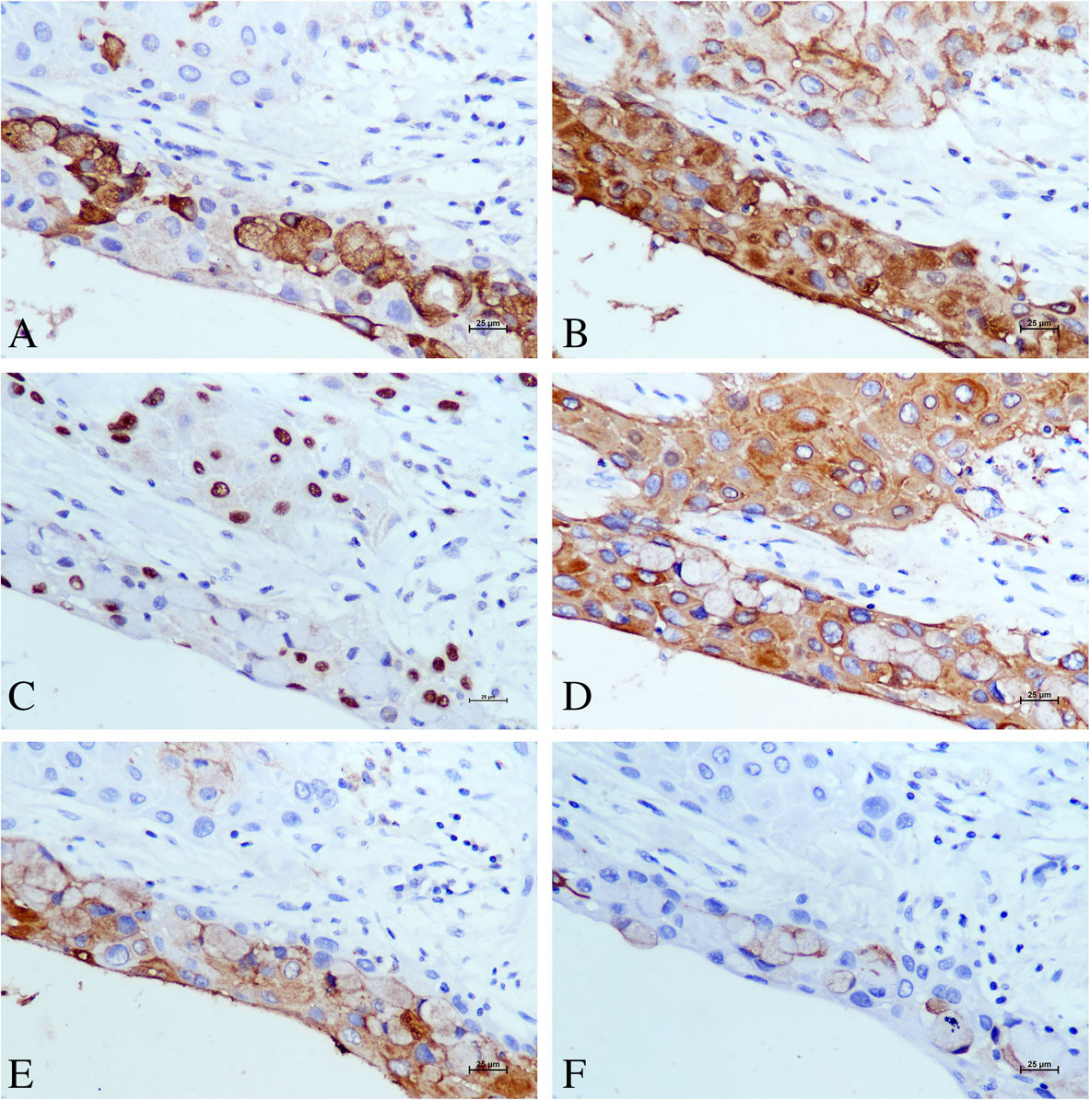
Immunohistochemical results. **a** Immunopositivity for cytoplasmic MUC5AC was detected in malignant mucinous cells, with negative staining in malignant epidermoid cells (magnification 400×). **b** Immunopositivity for membrane MUC1 was detected in all malignant epidermoid cells and mucinous cells (magnification 400×). **c** Immunopositivity for p63 was only detected in the nuclei of malignant epidermoid cells (magnification 400×). **d** Cytoplasmic CK19 staining was diffusely positive in the malignant epidermoid and mucous component of the tumor (magnification 400×). **e** Cytoplasmic CK7 staining was focally positive in the malignant epidermoid and mucous component of the tumor (magnification 400×). **f** Mucous component of the tumor was focally positive for membrane CEA staining (magnification 400×)

Immunohistochemically, MUC5AC was only positive in the cytoplasm of malignant mucous cells (Fig. 3a). Positive MUC1 was detected at the squamous and mucous tumor cell membrane (Fig. 3b). p63 was diffusely positive in the nuclei of malignant squamoid cells (Fig. 3c). Cytoplasmic CK19 was diffusely positive in malignant squamoid cells and mucous cells (Fig. 3d), and cytoplasmic CK7 was diffuse positive in malignant mucous cells (Fig. 3e). CEA was only focal positive in malignant mucous cells (Fig. 3f).

### FISH

**Fig. 4:**
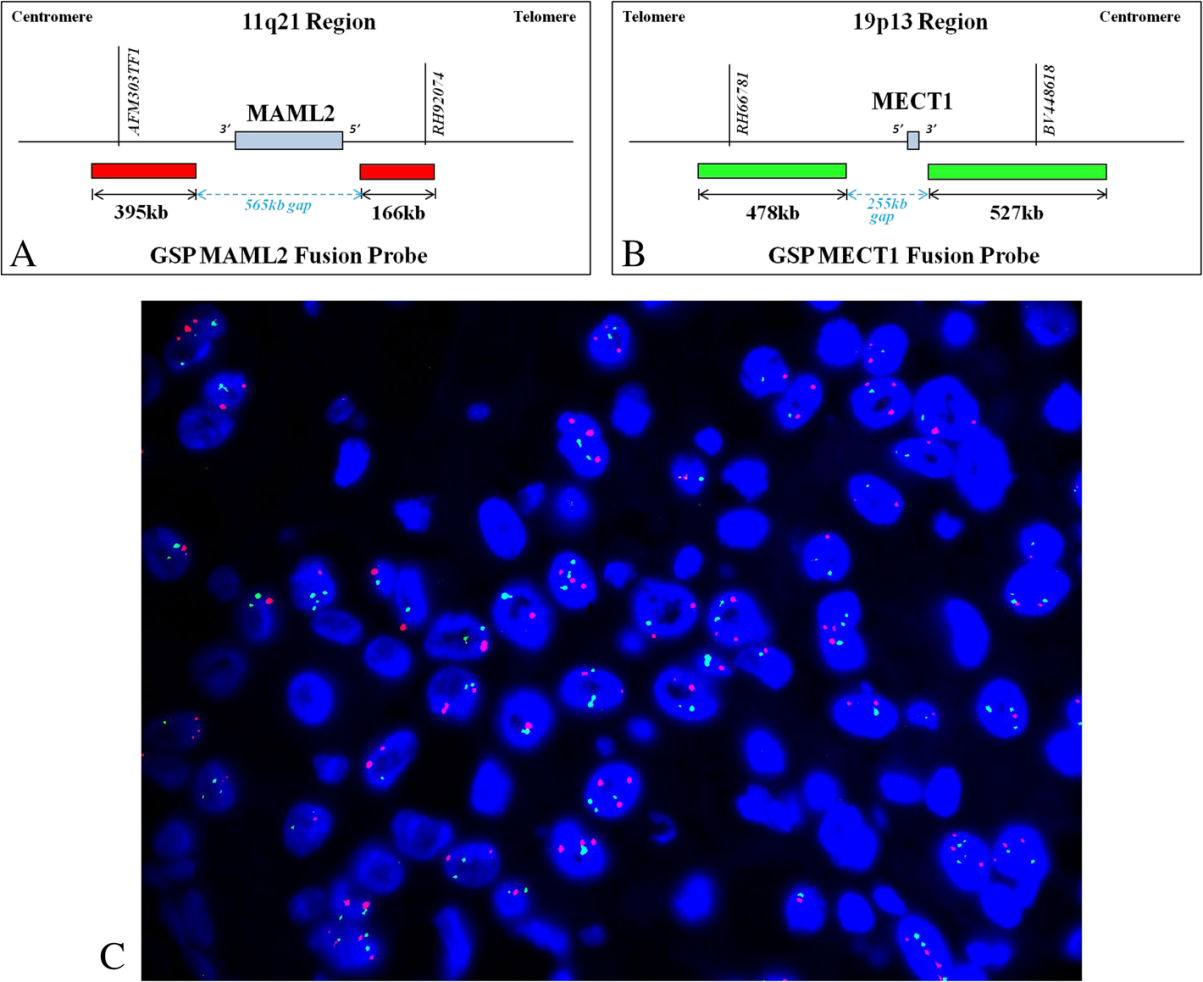
FISH analysis for the CRTC1/MECT1-MAML2 fusion gene. Probe pattern diagram: GSP CRTC1/MECT1 green fusion probe, GSP MAML2 red fusion probe (top). Non-typical positive signal mode, CRTC1/MECT1-MAML2 fusion gene was negative (bottom)

FISH analysis for the *CRTC1/MECT1-MAML2* fusion gene revealed negative results. Two hundred interphase cells were analyzed. The signal patterns were as follows: 2G2R, 7.5%; 3G2R, 40.0%; 3G1R, 5.0%; 4G3R, 10.0%; 4G2R, 5.0%; 4G4R, 7.5%; 2G3R, 5.0%; 3G4R, 5.0%; 2G1R, 5.0%; 3G3R, 7.5%; and 5G3R, 2.5% (Fig. 4).

### Somatic variants in the proband

**Fig. 5:**
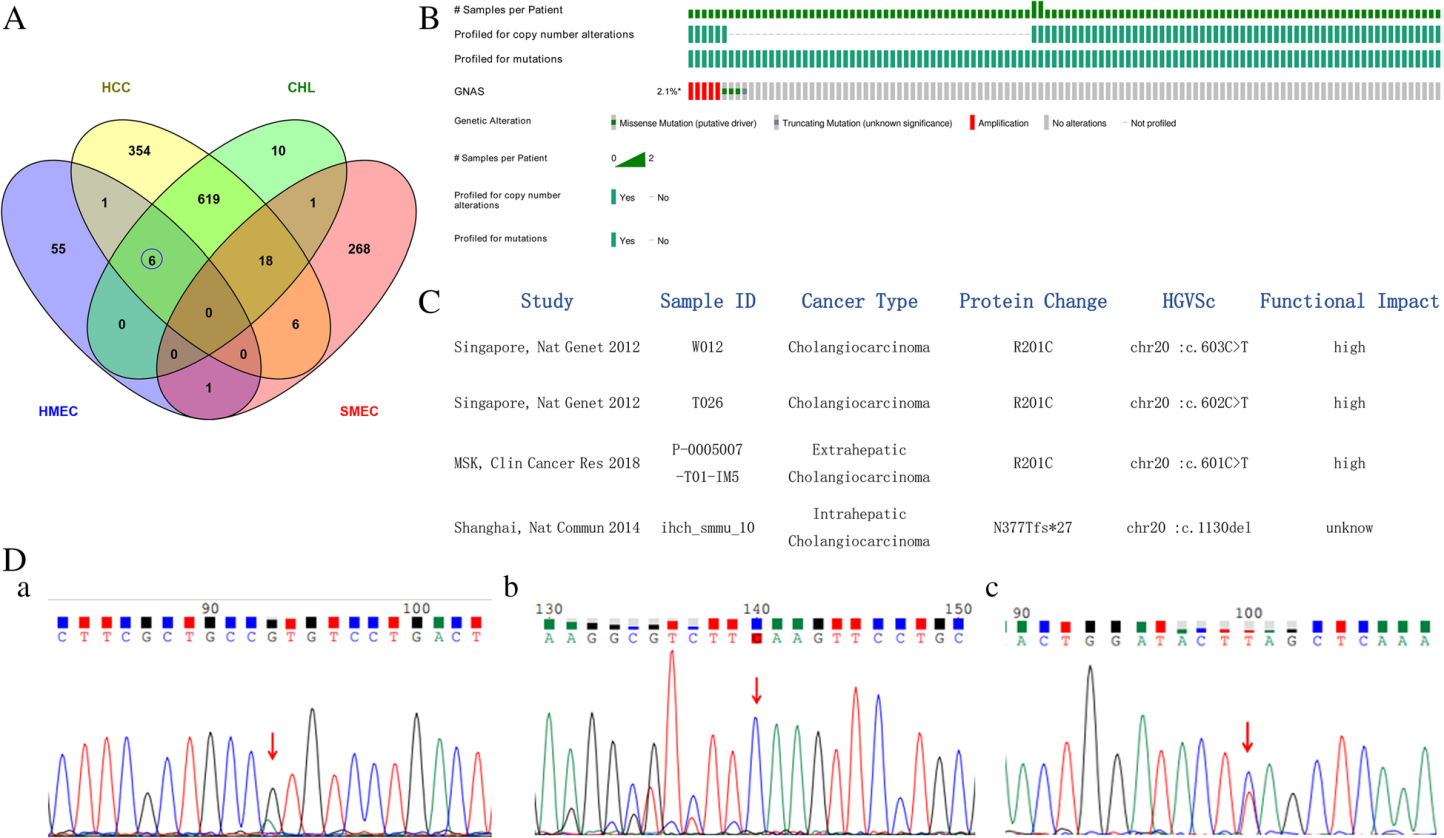
Public data analysis and significant somatic mutation in the proband. (Mutation diagram circles are colored with respect to the corresponding mutation types. In the case of different mutation types at a single position, the color of the circle reflects the most frequent mutation type). A. Venn diagram: six SNVs (in *STAT1*, *TGFBR1*, *NOTCH1*, *KMT2C*, *ELF3* and *GNAS* genes) in the HMEC overlapped with primary liver tumors (HCC and CHL). Only a SNV in the *CHD3* gene in HMEC overlapped with SMEC. B. Somatic *GNAS* gene mutation occurred in 2.1% (9/445) patients with primary hepatobiliary tumors in public databases. C. Three patients showed *GNAS* (p.R201H/C) missense mutation (putative driver). Samples (ID W012,T026) were O.pisthorchis viverrini-associated with cholangiocarcinoma (SRP007970)(*pentagonal shape*). D. Somatic mutations in the proband by Sanger sequencing: a. missense variant in *GNAS* (chr20.exon8:c.G602A:p.R201H), b. frameshift indel variant in *ELF3* (chr1,exon4:c.909dupC:p.F303fs), and c. nonsense variant in *KMT2C* (chr7,exon4:c.C1519T:p.Q507X) in tumor tissue

The sequencing of tumor tissue and corresponding non-tumor tissue in proband provided an average depth on target of 175X and 112X, respectively, and the >10X coverage of the targeted gene regions in tumor tissue and corresponding non-tumor tissue were 94 and 98%, respectively. The total somatic variants included 135 SNVs (53 missenses, 31 synonymous, 3 nonsense and others) and 4 InDels (2 frameshift insertions and 2 frameshift deletions). A total of 252 CNVs (126 gain, 126 loss) were identified in 2591 genes (629 gene gain, 1691 gene loss). *GNAS* mutation (NM_000516:exon8:c.G602A:p.R201H) was detected in both the tumor tissue and corresponding non-tumor tissue by WES, but the wild-type gene was determined in the corresponding non-tumor tissue by Sanger sequencing. In addition, protein-truncating genetic variants included a frameshift indel mutation in *ELF3* (c.909dupC:p.F303fs), and nonsense mutations in *DOCK3* (c.C2483A:p.S828X) and *KMT2C* (c.C1519T:p.Q507X) were detected in tumor samples by Sanger sequencing (Fig. 5D). A Circos map visually summarizes the somatic genomic variations of SNVs, INDEL and CNVs of the HMEC in Supplementary Figure 1.

### Bioinformatics analysis

A total of 1004 significant mutated genes (source: Onco KB) in SNVs from 8 studies containing 1487 HCC patients (Supplementary Fig. 2A) and 654 significant mutated genes (source: Onco KB) in SNVs from 7 studies containing 445 CHL patients were downloaded (Supplementary Fig. 2B) (www.cbioportal.org). We also obtained 312 significant mutated genes in SNVs from 18 SMEC patients from the study by Kang et al. Six SNVs (in *STAT1*, *TGFBR1*, *NOTCH1*, *KMT2C*, *ELF3* and *GNAS*) in the HMEC overlapped with those in primary liver tumors (HCC and CHL), and only an SNV in the *CHD3* gene in HMEC overlapped with SMEC (Fig. 5A). Somatic *GNAS* gene alterations (including missense mutation, amplification and truncating mutation) were detected in 2.1% (9/445) patients with primary hepatobiliary tumors including intrahepatic cholangiocarcinoma, extrahepatic cholangiocarcinoma and perihilar cholangiocarcinoma (Fig. 5B). Three patients showed *GNAS* (p.R201H/C) missense mutation (a putative driver) in exon 8. In addition, Sample ID (W012,T026) were *Opisthorchis viverrini*–associated cholangiocarcinoma (SRP007970) (Fig. 5C).

### Germline variants in the proband and pedigree analysis

**Fig. 6:**
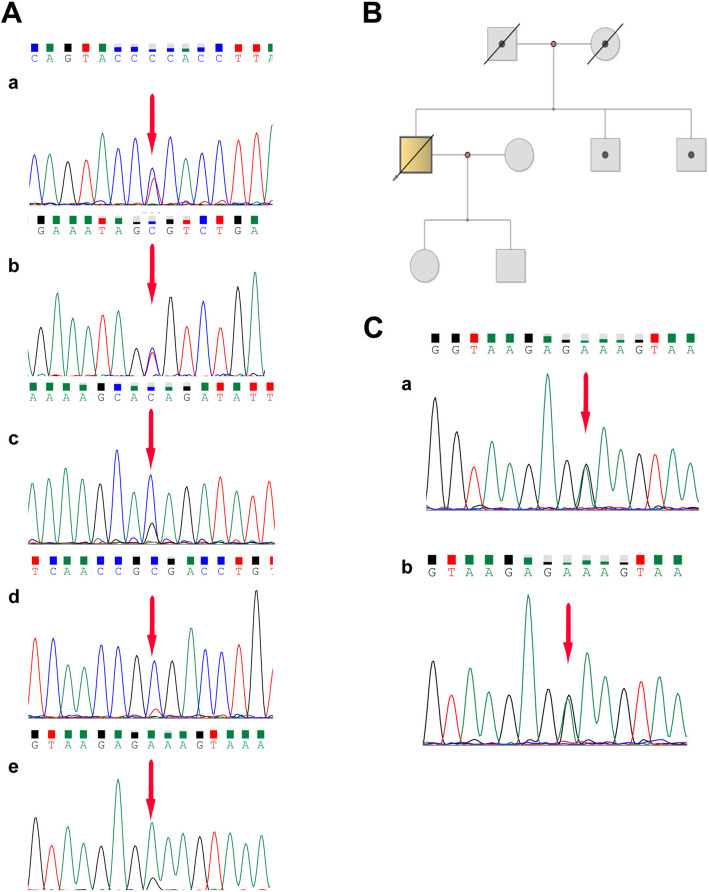
Germline heterozygous mutations in the proband and pedigree screening. A. a. *FANCI* (chr15.exon4:c.C257T:p.A86V); b. *FANCI* (chr15.exon22:c.G2225C:p.C742S); c. *FANCJ/BRIP1* (chr17.exon19:c.T2755C:p.S919P), d. *FANCA* (chr16.exon9:c.A796G:p.T266A); e. *FAN1* (chr15.exon2:c.G 698A:p.G233E) (corresponding non-tumor liver tissue). B. Pedigree map: the proband’s parents died from cardiovascular and cerebrovascular diseases; there was no obvious Fanconi’s anemia disease in the proband’s offspring and brothers. C. Siblings A and B, *FAN1* (exon2:c.G698A:p.G233E) (blood)

The 56 germline variants in 20 Fanconi’s anemia pathway genes included 19 missense variants, 25 synonymous variants and 12 UTR3 and UTR5 in the proband corresponding non-tumor tissue detected by WES (Supplementary Table 2). Five germline homozygous variants and six germline heterozygous variants in six Fanconi’s anemia pathway genes (*FANCA*, *FANCI*, *FANCW/RFWD3*, *FANCD1/BRCA2*, *FANCJ/BRIP1*, *FAN1*) were identified in the proband’s non-tumor tissue by Sanger sequencing (Table 2, Fig. 6A).

**Table 2:** The sanger suquencing of germline variants in proband and family members

Gene	Locus	HGVS nomenclature	Alt	Rff	Proband 64y	Sibling 70y	Sibling 61y	Son 39y	Daugther 41y
FANCI	15q26.1	exon4:c.C257T:p.A86V	CT	C	heterozygous	Non	Non	Non	Non
		exon22:c.G2225C:p.C742S	CG	C	heterozygous	Non	Non	Non	Non
FAN1	15q13.2	exon2:c.G698A:p.G233E	GA	G	heterozygous	heterozygous	heterozygous	Non	Non
FANCJ/BRIP1	17q23.2	exon19:c.T2755C:p.S919P	TC	T	heterozygous	homozygous	homozygous	homozygous	homozygous
FANCA	16q24.3	exon16:c.G1501A:p.G501S	CT	C	heterozygous	homozygous	homozygous	homozygous	homozygous
		exon9:c.A796G:p.T266A	TC	T	heterozygous	homozygous	homozygous	homozygous	homozygous
		exon26:c.G2426A:p.G809D	TT	C	homozygous	homozygous	homozygous	homozygous	homozygous
FANCW/RFWD3	16q23.1	exon10:c.A1690G:p.I564V	CC	T	homozygous	homozygous	homozygous	homozygous	homozygous
		exon2:c.C269A:p.T90N	TT	G	homozygous	homozygous	homozygous	homozygous	homozygous
FANCD1/BRCA2	13q13.1	exon14:c.T7397C:p.V2466A	GG	A	homozygous	homozygous	homozygous	homozygous	homozygous
C17orf70	17q25.3	exon8:c.A2449G:p.T817A	CC	T	homozygous	homozygous	homozygous	homozygous	homozygous

There was no family history of Fanconi’s anemia and solid tumor. The proband’s parents died of cardiovascular and cerebrovascular diseases 10 years ago. The older sibling showed hypertension and type 2 diabetes, and the proband’s offspring did not show any disease. The same mutational variants in *FANCD1/BRCA2*, *FANCW/RFWD3* and *FAP100/C17orf70* were verified in the pedigree. Heterozygous variants in *FANCA* (c.G1501A:p.G501S), *FANCA* (c.A796G:p.T266A) and *FANCJ/BRIP1* (c.T2755C:p.S919P) were detected in the proband, but homozygous mutations were shown in the other family members. Heterozygous FAN1 variants were detected in the siblings and proband. Only heterozygous *FANCI* variants (c.C257T:p.A86V and c.G2225C:p.C742S) were unique in the proband (Table 2, Fig. 6B, C).

## Discussion

The correlation between two rare diseases is really difficulty for clinicians. The emergence of next-generation sequencing technologies has provided a simple and powerful approach for discovering de novo disease-associated genes, and these methods have furthered our understanding of the rare HMEC, which is not associated with traditional high risk factors such as hepatitis B, hepatitis C, long-term alcohol intake history and cirrhosis, like most primary HMEC cases reported in the literature (Table 1). In 2006, Zhu et al. reported an adult male patient, also absent for high risk factors, who was first diagnosed with unusual HCC metastatic to the right proximal ulna and metachronous esophageal squamous cell carcinoma resulting from Fanconi’s anemia [24]. Linares et al. reported a 31-year-old female patient with synchronous squamous cell carcinoma of the esophagus and HCC associated with Fanconi’s anemia [25]. However, the present case only showed mild anemia, with an absence of the typical phenotype for Fanconi’s anemia, which was only present in family members carrying genes for Fanconi’s anemia. In fact, increasing evidence has suggested that monoallelic carriers for Fanconi’s anemia genes are characterized by an adult-onset predisposition to most solid cancers, especially squamous cell carcinomas from the epithelia of genitourinary tracts and the aerodigestive system [26]. Alter et al. reported that 20–30% of germline Fanconi’s anemia presents with unusual solid malignancies as the first clinic manifestation and shows an absence of the phenotype of congenital Fanconi’s anemia [26, 27]. Interestingly, three patients were reported with HMEC, also absent for Fanconi’s anemia, combined with synchronous HCC [28], synchronous squamous cell carcinoma in the cranial skin [29] and metachronous gastric malignant carcinoma [30].

The phenotypes of germline Fanconi’s anemia mutation–related solid tumors were probably determined in mutation loads or a dose-dependent manner [26]. So far, 22 proteins (FANCA—FANCW/RFWD3) and other Fanconi’s anemia network genes have been identified to function in the Fanconi’s anemia pathway [31]. These proteins participate in pathways including genome maintenance processes and DNA repair in response to DNA damage, as well as interstrand cross-linking repair, homologous recombination and non-homologous terminal junctions [32]. FANCA variants (c.G1501A:p.G501S and c.A796G:p.T266A) were shown to confer an increased risk for cervical squamous carcinoma [33]. An association with both progression-free and overall survival probabilities was found with the FANCD1/BRCA2 V2466A variant in breast cancer [34]. The Fanconi’s anemia protein FAN1 has a small role in interstrand crosslink repair and patients with homozygous mutation in *FAN1* did not show Fanconi’s anemia features. However, Lachaud et al. showed that FAN1 defects can cause cancers in knock-in mice, and the FAN1 variant in high-risk pancreatic cancers abolishes recruitment by Ub-FANCD2, resulting in genetic instability without affecting interstrand crosslink repair [35]. Germline *FAN1* mutations occur frequently in high-risk pancreatic cancers [36] and hereditary susceptibility to familial colorectal cancers [37]. In the present case, we found that *FAN1* mutations were present in the pedigree. Therefore, we propose that germline Fanconi’s anemia gene mutations are likely predisposing factors to the occurrence of HMEC.

Mutations in the *GNAS* gene typically occur at exon 8, in which Arg201 is converted to either a cysteine (R201C) or a histidine (R201H), leading to activation of the Gαs subunit. This Gαs constitutively activates the intracellular cyclin adenosine monophosphate signal pathway [38]. The GNAS R201H/C missense mutation have been showed a cross-communication between JAK/STAT and cyclic-AMP pathways in rare subtypes of liver inflammatory tumorigenesis [39]. Farges et al. also described an adult male patient with inflammatory hepatic adenoma associated with Fanconi’s anemia and somatic *GNAS* mutation who developed malignant transformation of HCC [40]. Consistent with the inflammatory phenotype observed in the present HMEC case, previous studies reported three HMEC patients with inflammation phenotype and inflammatory cell infiltration in the corresponding tumor-free liver tissue [30, 41, 42]. The somatic GNAS R201H/C mutation occurs frequently in secreting-mucous tumors like pancreatic intraductal papillary mucinous neoplasms (IPMN) as a bona fide precursor to carcinogenesis [43]. The GNAS R201 mutation was detected in pancreatic IPMN tissue, secretin-stimulated pancreatic juice and peripheral blood [44]. The mucus production and carcinogenesis of the intestinal subtype of intraductal papillary neoplasm of the biliary ducts, a counterpart of pancreatic IPMN, have also been connected with gain-of-function mutations of GNAS R201 [45]. The protein-truncating genetic variants in the present case included a frameshift insertion in *ELF3* and nonsense mutation in *KMT2C*, and both genes were also reported in biliary tumors as tumor suppressors [46]. In addition, positive immunohistochemical results for CK7 and CK19 were detected in the present case, which suggests the possibility that HMEC originates from the biliary system [43]. Moreover, GNAS and KMT2C were almost only mutated in *Opisthorchis viverrini*–induced CHL compared non-*O. viverrini*–induced CHL [47, 48], which is consistent with the study by Jarley et al. showing that HMEC may be induced in three patients by biliary parasite infection [49]. This may also explain the observation that most HMECs (20/23) were reported in Asian areas with a high incidence of parasites (Table 1).

We also found that the *CRTC1-MAML2* fusion gene, which is a specific driver mutation in SMEC, was negative in the present case as examined by FISH. The *CRTC1-MAML2* translocation is found in 50–60% of all SMECs, with the low- and intermediate-grades having a higher percentage of translocations [50]. The present case showed an admixture of malignant epidermoid and mucin-secreting cells in lack of gland formations and keratinization is rarely formed in malignant epidermoid cells, thus favoring a diagnosis of HMEC rather than primary hepatic adenosquamous carcinoma. Immunohistochemical evaluation of the present case also favored HMEC; CEA was focally positive in malignant mucinous cells, while it tends to show diffuse positive staining in primary hepatic adenosquamous carcinoma cells [51].

In conclusion, the molecular characteristics of the current HMEC were more similar to primary liver biliary tumors rather than to SMEC. We present here for the first time the etiology of HMEC associated with germline Fanconi’s anemia mutations and somatic GNAS R201 mutation.

## Supplementary Information

**Additional file 1: Supplementary Figure 1** Somatic variation Circos plot display. **Supplementary Figure 2.** Public datas (source: Onco KB). **Supplementary Table 1.** The Primers of somatic and germline mutations. **Supplementary Table 2.** The germline variants in Fanconi’s anemia pathway genes in the proband’s corresponding non-tumor tissue by WES.

## Data Availability

We declared that materials described in the manuscript, including all relevant raw data, will be freely available to any scientist wishing to use them for non-commercial purposes, without breaching participant confidentiality.

## Abbreviations

MEC: Mucoepidermoid carcinoma
HMEC: Hepatic mucoepidermoid carcinoma
SMEC: Salivary mucoepidermoid carcinoma
HCC: Hepatocellular carcinoma
CHL: Cholangiocarcinoma
WES: Whole exome-sequencing
FISH: Fluorescence in situ hybridization
CT: Computer tomography
SNV: Single-nucleotide variant
