# Supplementary material for: Whole-exome sequencing reveals the etiology of the rare primary hepatic mucoepidermoid carcinoma
Source: Diagn Pathol. 2021 Apr 8;16:29. doi: 10.1186/s13000-021-01086-3 (PMC8034126; doi:10.1186/s13000-021-01086-3)
Supplement: Supplementary file 1 — Additional file 1: Supplementary Figure 1 Somatic variation Circos plot display. Supplementary Figure 2. Public datas (source: Onco KB). Supplementary Table 1. The Primers of somatic and germline mutations. Supplementary Table 2. The germline variants in Fanconi’s anemia pathway genes in the proband’s corresponding non-tumor tissue by WES. [file 13000_2021_1086_MOESM1_ESM.docx]

#### Supplementary materials

**Supplementary Figure 1:**The first circle indicates the sequence coverage diagram; in the second circle, the green dot indicates the density of SNP indel; and the third circle shows CNV result. Red indicates a gain in the number of copies, blue indicates loss in number of copies, and green indicates neutral number of copies.

**
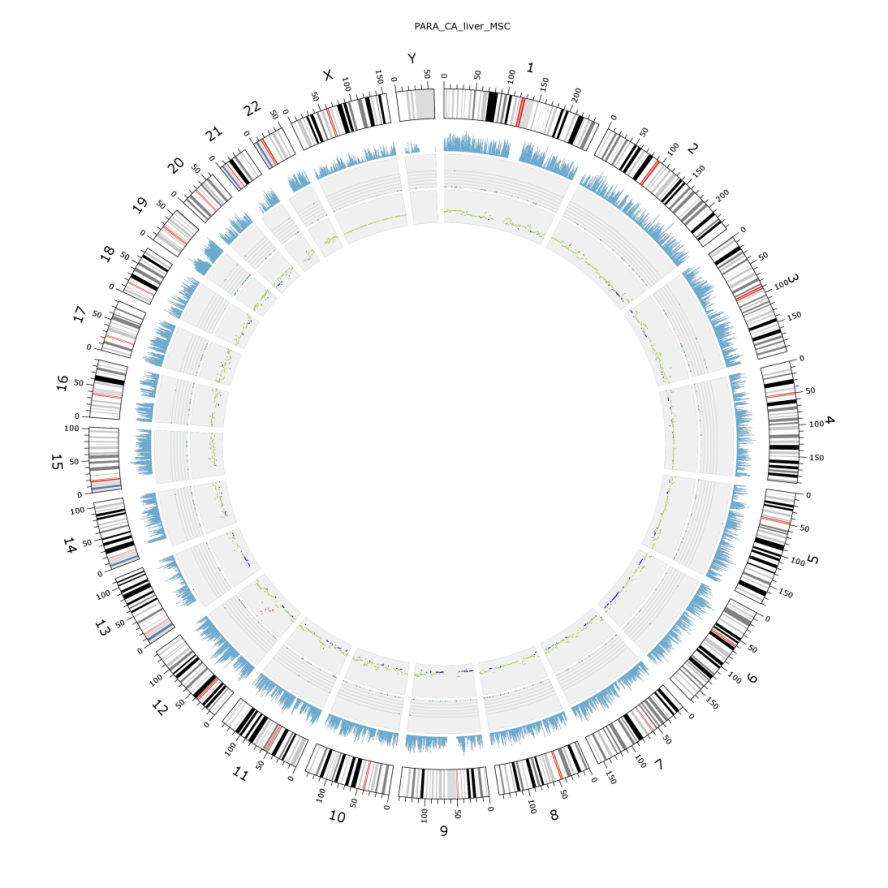
**

**Supplementary figure 2 A.** 8 studies contained 1487 HCC patients **B:** 7 studies contained 445 CHL patients (source :Onco KB ).


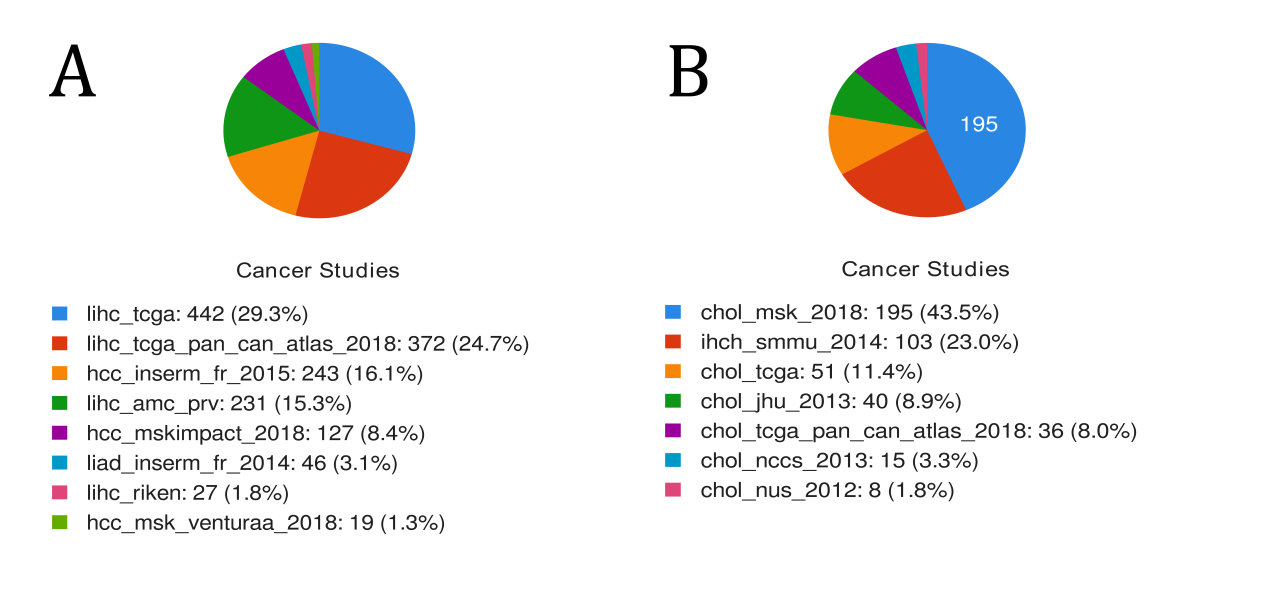


**Supplementary table 1*:***The Primers of somatic and germline mutations

| **Genes** | **HVPG** | **Position** | **Forward amplification primer** | **Reverse amplification primer** | **Bp** | **Sequencing primer sequence** |
| --- | --- | --- | --- | --- | --- | --- |
| **GNAS** | GNAS:NM_001077489:exon7:c.G557A:p.R186H\|GNAS:NM_080426:exon7:c.G560A:p.R187H\|GNAS:NM_000516:exon8:c.G602A:p.R201H\|GNAS:NM_001077488:exon8:c.G605A:p.R202H\|GNAS:NM_080425:exon8:c.G2531A:p.R844H | rs57484421 | TCAAGCAGGCTGACTATGTG | ACTGGGGTGAATGTCAAGAA | 300 | ACTCTGAGCCCTCTTTCCAA |
| **BCL2** | NM_000657:exon2:c.G298A:p.A100T | rs60985602 | CGCAGAGGGGCTACGA | CCCAGACTCACATCACCAA | 544 | CACCGTGGCAAAGCGTCCCC |
| **ELF3** | ELF3:NM_004433:exon8:c.909dupC:p.F303fs | rs201983059 | CTGGATGGCAAACTGATGGA | CACAGGCGATACAAGAAATG | 471 | TCTGCTGTCTGCCAGGCTAATA |
| **DOCK3** | DOCK3:NM_004947:exon24:c.C2483A:p.S828X | rs51308373 | CACCACCTTGTATCATTGTC | TTATCACTTTGCTATCACCC | 356 | ACACCCTAACTCTGTGCTAACC |
| **KMT2C** | KMT2C:NM_170606:exon11:c.C1519T:p.Q507X | rs151949126 | AGAAATGAGCACTAATAGGGAA | TGGTGATACAATGGCACAAC | 467 | GCTTTTCAGCAATGCCTTCT |
| **SLC39A4** | SLC39A4:NM_130849:exon4:c.G739T:p.G247X | rs145640423 | TCTTGCTTCCACGCCTTGC | AGCCTGTTCCGACAGTCCAT | 472 | GCGGTGCCAACTCTTCTCC |
| **SLX4** | SLX4:NM_032444:exon12:c.C3365T:p.P1122L | rs714181 | ATAACAAGTGAGCCCGAGG | TGGACTTCATTTTGGTTTG | 514 | GTGCCATCAAAGCAGAAAAGG |
| **RFWD3** | RFWD3:NM_018124:exon10:c.A1690G:p.I564V | rs7193541 | TGACTAACTGTGGAACACC | AGAACTCAAGAGGAAAACC | 491 | GAGTTACCTGGGCAGATTC |
| **RFWD3** | RFWD3:NM_018124:exon2:c.C269A:p.T90N | rs8058922 | TGGCAGAGCCTCAGTTTCA | GAAGTGGAGGTCTTGGGAG | 526 | GGCTCTTGCCCTCCGTGAA |
| **FANCM** | FANCM:NM_020937:exon2:c.C524T:p.S175F | rs10138997 | TTTCCTGGCATTCTTCCTGA | TTATTTCAGCAGCGGGACAA | 408 | TTGTAGTCCTAGATAAGTGCC |
| **FANCM** | FANCM:NM_020937:exon14:c.G2632T:p.V878L | rs1367580 | CTGATGAAGAATGTGCTGAA | ACAGGGTAAGAAAAGCAAAT | 581 | AGGCGGAGGAGAATAAGATA |
| **FANCM** | FANCM:NM_020937:exon16:c.A4378G:p.I1460V | rs78211950 | TAGATTGTTATGATTGGGTA | ATACATCTTTGAAAACTAACA | 511 | TACCTTGGGCTTCTGCTGAG |
| **FANCM** | FANCM:NM_020937:exon21:c.C5434G:p.P1812A | rs3736772 | AAGAGTTTCCCACATCCTAA | GAAGGACGAAAACTGGACA | 583 | TACTCACGATGTAATCACAG |
| **FANCI** | FANCI:NM_001113378:exon4:c.C257T:p.A86V,FANCI:NM_018193:exon4:c.C257T:p.A86V | rs17803620 | ATTTTAGTCCCTTTATTTTGGC | TCAAGCGATCCTCACCTCA | 529 | AGCACCGTAGTAATCAGTCG |
| **FANCI** | FANCI:NM_001113378:exon22:c.G2225C:p.C742S,FANCI:NM_018193:exon22:c.G2225C:p.C742S | rs2283432 | CAGTGTCATCGTCTGTGGT | TGTATTCAGCATTGTTTGG | 472 | AATACAGTCATACCCTTACA |
| **FANCD2** | FANCD2:NM_001018115:exon23:c.C2141T:p.P714L,FANCD2:NM_033084:exon23:c.C2141T:p.P714L | rs3864017 | GCCTCCCAGGTTCAAGCAAT | TCTCCGCAGTGACTTTCCAT | 501 | CTCCCTAACCTCTCCAAAT |
| **FANCD2** | FANCD2:NM_001018115:exon29:c.C2723T:p.T908I,FANCD2:NM_033084:exon29:c.C2723T:p.T908I | rs188375397 | GTGCTCTGTCTTGGGAATA | CCTAGCCTGTCACTTACTTC | 332 | TCAGTATCTAAGATGAACTTCG |
| **FANCA** | FANCA:NM_000135:exon26:c.G2426A:p.G809D,FANCA:NM_001286167:exon26:c.G2426A:p.G809D | rs7195066 | GGTTATCTTTGGGTGGTATG | CAGGCTGCTACTTCTCAAT | 393 | CCACCCTCATTCTCGTTGC |
| **FANCA** | FANCA:NM_000135:exon16:c.G1501A:p.G501S,FANCA:NM_001286167:exon16:c.G1501A:p.G501S | rs2239359 | CCGAGGCAAGACCAGACAT | GTCCCAGGCAGTTCCCAGA | 419 | AGGGCTCAAGCAACATTACC |
| **FANCA** | FANCA:NM_000135:exon9:c.A796G:p.T266A,FANCA:NM_001018112:exon9:c.A796G:p.T266A,FANCA:NM_001286167:exon9:c.A796G:p.T266A | rs7190823 | TGCTTCCCTGTGACCCA | GTGCCGTTTCTTTACTTTAG | 345 | ACCTCAAATGGAAAGGCAGAA |
| **FAN1** | FAN1:NM_001146094:exon2:c.G698A:p.G233E,FAN1:NM_001146095:exon2:c.G698A:p.G233E,FAN1:NM_001146096:exon2:c.G698A:p.G233E,FAN1:NM_014967:exon2:c.G698A:p.G233E | rs4779794 | TTTGCCGGTTCTAGTCCACA | TGCAGAACTATGAGATTTTGCCT | 456 | AAGAGCCTGATTGATAACT |
| **ERCC6L2** | ERCC6L2:NM_001010895:exon1:c.A1G:p.M1V | rs690528 | GCTTGGGTCCCCTTAGTCG | GCCTGGAGGGGTTCACAAT | 385 | CTCCTCCATCCTGTGGCTTCG |
| **C17orf70** | C17orf70:NM_025161:exon8:c.A2449G:p.T817A | rs14422 | GGACCCACCACTCGCAGGAC | AGGCAGGGAGGCAGCACCAG | 488 | CTCTGCGGACCCCGAGTGA |
| **BRIP1** | BRIP1:NM_032043:exon19:c.T2755C:p.S919P | rs4986764 | GCCCTACTTCTACTGCCTCT | CTGGAGATAATGCTACTTGG | 569 | AAATGGGTACGGCAGCAGA |
| **BRCA2** | BRCA2:NM_000059:exon14:c.T7397C:p.V2466A | rs169547 | ATTCCAGTAACAGCAGTCC | ACTTGGAGGAAAACAGACA | 397 | TTAAAATTACCACCACCAA |

**Supplementary table 2**:The germline variants in Fanconi’s anemia pathway genes in the proband’s corresponding non-tumor tissue by WES

| **Gene** | **CytoBand** | **REF** | **ALT** | **ID** | **ExonicFunc** | **AAChange** |
| --- | --- | --- | --- | --- | --- | --- |
| FANCL | 2p16.1 | A | G | rs848291 | synonymous SNV | NM_001114636:exon12:c.T996C:p.S332S,  NM_018062:exon12:c.T981C:p.S327S |
| FANCD2 | 3p25.3 | A | G | rs34046352 | synonymous SNV | NM_001018115:exon14:c.A1122G:p.V374V,  NM_033084:exon14:c.A1122G:p.V374V |
| FANCD2 | 3p25.3 | C | T | rs3864017 | missense SNV | NM_001018115:exon23:c.C2141T:p.P714L,  NM_033084:exon23:c.C2141T:p.P714L |
| FANCD2 | 3p25.3 | C | T | rs188375397 | missense SNV | NM_001018115:exon29:c.C2723T:p.T908I,  NM_033084:exon29:c.C2723T:p.T908I |
| FANCD2 | 3p25.3 | T | G | rs2272125 | synonymous SNV | NM_001018115:exon42:c.T4098G:p.L1366L,  NM_033084:exon42:c.T4098G:p.L1366L |
| FANCE | 6p21.31 | A | C | rs4713867 | synonymous SNV | NM_021922:exon2:c.A387C:p.P129P |
| FANCM | 14q21.2 | C | T | rs10138997 | missense SNV | NM_020937:exon2:c.C524T:p.S175F |
| FANCM | 14q21.2 | G | T | rs1367580 | missense SNV | NM_020937:exon14:c.G2632T:p.V878L |
| FANCM | 14q21.2 | A | G | rs78211950 | missense SNV | NM_020937:exon16:c.A4378G:p.I1460V |
| FANCM | 14q21.2 | C | G | rs3736772 | missense SNV | NM_020937:exon21:c.C5434G:p.P1812A |
| FANCI | 15q26.1 | C | T | rs17803620 | missense SNV | NM_001113378:exon4:c.C257T:p.A86V,  NM_018193:exon4:c.C257T:p.A86V |
| FANCI | 15q26.1 | G | C | rs2283432 | missense SNV | NM_001113378:exon22:c.G2225C:p.C742S,  NM_018193:exon22:c.G2225C:p.C742S |
| FANCI | 15q26.1 | G | A | rs7183618 | synonymous SNV | NM_001113378:exon24:c.G2547A:p.K849K |
| FANCI | 15q26.1 | T | C | rs1138465 | synonymous SNV | NM_018193:exon36:c.T3726C:p.G1242G,  NM_001113378:exon37:c.T3906C:p.G1302G |
| SLX4 | 16p13.3 | A | G | rs3810812 | synonymous SNV | NM_032444:exon12:c.T4500C:p.N1500N |
| SLX4 | 16p13.3 | G | A | rs714181 | missense SNV | NM_032444:exon12:c.C3365T:p.P1122L |
| SLX4 | 16p13.3 | C | T | rs76488917 | synonymous SNV | NM_032444:exon12:c.G3162A:p.S1054S |
| SLX4 | 16p13.3 | G | A | rs28516461 | synonymous SNV | NM_032444:exon3:c.C678T:p.H226H |
| ERCC4 | 16p13.12 | T | C | rs1799801 | synonymous SNV | NM_005236:exon11:c.T2505C:p.S835S |
| FANCA | 16q24.3 | C | T | rs7195066 | missense SNV | NM_000135:exon26:c.G2426A:p.G809D,  NM_001286167:exon26:c.G2426A:p.G809D |
| FANCA | 16q24.3 | C | T | rs2239359 | missense SNV | NM_000135:exon16:c.G1501A:p.G501S,  NM_001286167:exon16:c.G1501A:p.G501S |
| FANCA | 16q24.3 | T | C | rs7190823 | missense SNV | NM_000135:exon9:c.A796G:p.T266A,  NM_001018112:exon9:c.A796G:p.T266A,  NM_001286167:exon9:c.A796G:p.T266A |
| ERCC6L2 | 9q22.32 | A | G | rs690528 | missense SNV | NM_001010895:exon1:c.A1G:p.M1V |
| BRCA2 | 13q13.1 | A | G | rs1801406 | synonymous SNV | NM_000059:exon11:c.A3396G:p.K1132K |
| BRCA2 | 13q13.1 | T | C | rs543304 | synonymous SNV | NM_000059:exon11:c.T3807C:p.V1269V |
| BRCA2 | 13q13.1 | A | G | rs206075 | synonymous SNV | NM_000059:exon11:c.A4563G:p.L1521L |
| BRCA2 | 13q13.1 | G | C | rs206076 | synonymous SNV | NM_000059:exon11:c.G6513C:p.V2171V |
| BRCA2 | 13q13.1 | A | G | rs1799955 | synonymous SNV | NM_000059:exon14:c.A7242G:p.S2414S |
| BRCA2 | 13q13.1 | T | C | rs169547 | missense SNV | NM_000059:exon14:c.T7397C:p.V2466A |
| BRIP1 | 17q23.2 | A | G | rs4986763 | synonymous SNV | NM_032043:exon20:c.T3411C:p.Y1137Y |
| BRIP1 | 17q23.2 | A | G | rs4986764 | missense SNV | NM_032043:exon19:c.T2755C:p.S919P |
| BRIP1 | 17q23.2 | T | C | rs4986765 | synonymous SNV | NM_032043:exon19:c.A2637G:p.E879E |
| UBE2T | 1q32.1 | T | C | rs14451 | synonymous SNV | NM_014176:exon2:c.A15G:p.S5S |
| RFWD3 | 16q23.1 | T | C | rs7193541 | missense SNV | NM_018124:exon10:c.A1690G:p.I564V |
| RFWD3 | 16q23.1 | A | T | rs7188880 | synonymous SNV | NM_018124:exon10:c.T1623A:p.P541P |
| RFWD3 | 16q23.1 | C | T | rs4888262 | synonymous SNV | NM_018124:exon8:c.G1212A:p.T404T |
| RFWD3 | 16q23.1 | G | T | rs8058922 | missense SNV | NM_018124:exon2:c.C269A:p.T90N |
| C17orf70 | 17q25.3 | T | C | rs14422 | missense SNV | NM_025161:exon8:c.A2449G:p.T817A |
| C17orf70 | 17q25.3 | A | G | rs8077430 | synonymous SNV | NM_025161:exon7:c.T2322C:p.T774T |
| APITD1-CORT | 1p36.22 | C | T | rs628462 | synonymous SNV | NM_001302:exon2:c.C210T:p.A70A;  NM_001243768:exon4:c.C324T:p.A108A;  NM_198544:exon5:c.C387T:p.A129A |
| APITD1-CORT | 1p36.22 | T | C | rs666103 | synonymous SNV | NM_001302:exon2:c.T255C:p.S85S;  NM_001243768:exon4:c.T369C:p.S123S,  NM_198544:exon5:c.T432C:p.S144S |
| FAN1 | 15q13.2 | G | A | rs4779794 | missense SNV | NM_001146094:exon2:c.G698A:p.G233E,  NM_001146095:exon2:c.G698A:p.G233E,  NM_001146096:exon2:c.G698A:p.G233E,  NM_014967:exon2:c.G698A:p.G233E |
| FAN1 | 15q13.3 | T | C | rs2955795 | synonymous SNV | NM_014967:exon14:c.T3015C:p.H1005H |
| UBE2T | 1q32.1 | T | C | rs14451 | synonymous SNV | NM_014176:exon2:c.A15G:p.S5S |
